# Correspondence Between Genomic- and Genealogical/Coalescent-Based Inference of Homozygosity by Descent in Large French-Canadian Genealogies

**DOI:** 10.3389/fgene.2021.808829

**Published:** 2022-01-21

**Authors:** Kelly M. Burkett, Mohan Rakesh, Patricia Morris, Hélène Vézina, Catherine Laprise, Ellen E. Freeman, Marie-Hélène Roy-Gagnon

**Affiliations:** ^1^ Department of Mathematics and Statistics, University of Ottawa, Ottawa, ON, Canada; ^2^ School of Epidemiology and Public Health, University of Ottawa, Ottawa, ON, Canada; ^3^ Projet BALSAC, Université du Québec à Chicoutimi, Chicoutimi, QC, Canada; ^4^ Département des Sciences Humaines et Sociales, Université du Québec à Chicoutimi, Chicoutimi, QC, Canada; ^5^ Centre Intersectoriel en Santé Durable, Université du Québec à Chicoutimi, Chicoutimi, QC, Canada; ^6^ Département des Sciences Fondamentales, Université Du Québec à Chicoutimi, Chicoutimi, QC, Canada; ^7^ Centre de Recherche, Hĉpital Maisonneuve-Rosemont, Montréal, QC, Canada

**Keywords:** homozygosity by descent, founder populations, genealogical data, coalescent models, most recent common ancestor, simulations

## Abstract

Research on the genetics of complex traits overwhelmingly focuses on the additive effects of genes. Yet, animal studies have shown that non-additive effects, in particular homozygosity effects, can shape complex traits. Recent investigations in human studies found some significant homozygosity effects. However, most human populations display restricted ranges of homozygosity by descent (HBD), making the identification of homozygosity effects challenging. Founder populations give rise to higher HBD levels. When deep genealogical data are available in a founder population, it is possible to gain information on the time to the most recent common ancestor (MRCA) from whom a chromosomal segment has been transmitted to both parents of an individual and in turn to that individual. This information on the time to MRCA can be combined with the time to MRCA inferred from coalescent models of gene genealogies. HBD can also be estimated from genomic data. The extent to which the genomic HBD measures correspond to the genealogical/coalescent measures has not been documented in founder populations with extensive genealogical data. In this study, we used simulations to relate genomic and genealogical/coalescent HBD measures. We based our simulations on genealogical data from two ongoing studies from the French-Canadian founder population displaying different levels of inbreeding. We simulated single-nucleotide polymorphisms (SNPs) in a 1-Mb genomic segment from a coalescent model in conjunction with the observed genealogical data. We compared genealogical/coalescent HBD to two genomic methods of HBD estimation based on hidden Markov models (HMMs). We found that genomic estimates of HBD correlated well with genealogical/coalescent HBD measures in both study genealogies. We described generation time to coalescence in terms of genomic HBD estimates and found a large variability in generation time captured by genomic HBD when considering each SNP. However, SNPs in longer segments were more likely to capture recent time to coalescence, as expected. Our study suggests that estimating the coalescent gene genealogy from the genomic data to use in conjunction with observed genealogical data could provide valuable information on HBD.

## 1 Introduction

Inbreeding leads to increased homozygosity and has a negative effect on phenotypes (Charlesworth and Willis, 2009). This phenomenon is referred to as inbreeding depression and is well documented in plants and animals (Roff, 1997). In humans, inbreeding depression has long been reported from small-scale pedigree studies (Lebel and Gallagher, 1989; Shami et al., 1991; Scriver, 2001), but the quantification of inbreeding depression effects on human phenotypes from these studies is limited. More recently, the availability of large study samples with genome-wide genotypic data has allowed the detection of homozygosity effects on a wide range of phenotypes and has also allowed some quantification of the effect of inbreeding depression on human phenotypic variation (Joshi et al., 2015; Zhu et al., 2015; Johnson et al., 2018; Clark et al., 2019; Yengo et al., 2021). However, most human populations display restricted ranges of homozygosity by descent (HBD), making the identification and quantification of the effect of inbreeding depression challenging in humans (Keller et al., 2011; Yengo et al., 2021).

Founder populations give rise to higher HBD levels. The French-Canadian founder population originated at the beginning of the 17th century with the immigration of French settlers (Charbonneau et al., 1993), which ended in 1759 after the British conquest. The French-Canadian population expanded rapidly and was relatively isolated because of linguistic, religious, and geographic barriers (Bouchard and De Braekeleer, 1990). Approximately 8,500 founders contributed to the genetic background of the French-Canadian founder population (Charbonneau et al., 2000). As population size grew, new regions of Quebec were settled, including remote and isolated regions, which resulted in population structure (Roy-Gagnon et al., 2011). In the isolated region of Saguenay–Lac-Saint-Jean (SLSJ), French-Canadian settlement was initiated around 1840 by inhabitants of the neighboring region of Charlevoix, and until about 1910, 75% of the 30,000 immigrants to Saguenay came from Charlevoix (Pouyez et al., 1983). In contrast, urban regions like the Montreal region saw more diverse immigration patterns (McInnis, 2000; Piché, 2003), including migration from other regions of Quebec and more mixing.

The probability of an individual's allele being HBD within a fixed genealogy depends on the number of meiosis on the transmission paths of the individual's two copies of the alleles through a specific most recent common ancestor (MRCA) and also on all the possible paths linking those two alleles through this common ancestor. When the MRCA occurs further back in time than the founders of a fixed pedigree, time to MRCA for two genomic segments is available from the gene genealogy (Hudson, 1990), which describes the relationships between genomic segments sampled at present. The gene genealogy cannot be observed but can be modeled using the coalescent (Kingman, 1982). The gene genealogy differs from a family tree by tracking the descent of genetic material in a genomic region rather than an individual's actual ancestors. In the presence of recombination, the gene genealogy can be described by a set of trees with each giving the ancestral history of the sample at a locus in the region. In study samples from founder populations with deep genealogical data available, it is possible to use both the study and coalescent genealogy to gain information on the time to the MRCA from whom a chromosomal segment has been transmitted to both parents of an individual and in turn to that individual. It is then possible to describe the complex patterns of relatedness, present in a founder population like the French-Canadians, that give rise to a wide range of identity-by-descent sharing of chromosomal segments arising from a large number of complex ancestral sharing paths (Gauvin et al., 2014; Gauvin et al., 2015).

Sharing of chromosomal segments within individuals can be observed with genomic data. HBD can then be estimated by searching for runs of homozygosity (ROHs) exceeding a given length (e.g., 1,000 or 1,500 kb) along a genomic region (McQuillan et al., 2008; Howrigan et al., 2011). Alternatively, HBD can be inferred by modeling the HBD states of each genomic marker in the region using hidden Markov models (HMMs) (Leutenegger et al., 2003; Han and Abney, 2011; Han and Abney, 2013; Gazal et al., 2014a). HMM-based approaches that formally model identity-by-descent sharing may perform better to capture complex relatedness in founder populations. The extent to which genomic inference of HBD captures sharing through complex ancestral paths in founder populations has not been studied with simulations using complex genealogical structures. In this study, we aimed to describe the relationship between genomic estimates of HBD probability and HBD probability from simulated genealogies. We used coalescent models in conjunction with extended genealogical data collected by two ongoing studies conducted in the French-Canadian founder population to simulate genomic segments passed down to individuals from the two studies. From these simulations, we assessed the correspondence between two genomic estimates of HBD from two HMM-based methods (IBDLD and FEstim) and the relationship between these estimates and the time to MRCA from the gene genealogy observed in the simulations.

## 2 Materials and Methods

### 2.1 Simulations

Simulations were based on large genealogies from the French-Canadian founder population. These genealogies come from two studies with different designs: a hospital-based cross-sectional study of eye disease and cognitive phenotypes conducted at the ophthalmology clinics of Maisonneuve-Rosemont Hospital in Montreal (Varin et al., 2017; Varin et al., 2020) and a family study of asthma from the SLSJ region (Laprise, 2014). In the Montreal study, unrelated (to the investigators' knowledge) participants with either glaucoma, age-related macular degeneration (AMD) or normal vision were recruited. Families in the SLSJ study were recruited through probands with a diagnosis of asthma. Genealogies were obtained from the BALSAC database (BALSAC project, Université du Québec à Chicoutimi, https://balsac.uqac.ca/). BALSAC contains over 4.3 million records providing information on over 6 million individuals and allowing the reconstruction of ascending genealogies from present-day individuals going back over four centuries (Vézina and Bournival, 2020). Participants from the two studies provided information on the names and location of marriage of their recent ancestors, which allowed their genealogies to be reconstructed in BALSAC. A subset of the present-day individuals with French-Canadian ancestry was selected from the Montreal study as probands for the simulations. A similar number of probands was selected from the SLSJ genealogies (all of French-Canadian ancestry) for comparison purposes.

Haplotypes for the founders of the genealogies were simulated from a coalescent model using the ms (Hudson, 2002) algorithm implemented in the phyclust R package version 0.1–30 (Chen, 2011). Simulated haplotypes covered a 1 Mb region. The recombination and mutation rates for the coalescent model were both set to be 10^–8^, and the effective population size was set to 10,000. In addition to the haplotypes of the founders, the ms algorithm returns all gene genealogies for the given genomic region. We then used a new simulation function that we implemented in the GENLIB R package version 1.1.5 (Gauvin et al., 2015). GENLIB is specifically designed to analyze large genealogical datasets and has several functionalities including genealogical data management, descriptive statistics, and simulations. The new simulation function, “gen.simuhaplo,” can be used to pass down haplotypes from founders to selected individuals (probands) through the genealogy. At each meiosis, recombination is simulated using the no-chromosome interference model of meiosis; the crossover events are modelled as a homogeneous Poisson process, with user-specified recombination rates for males and females. For our simulations, given the 1 Mb segments being passed down from the founders, we used a recombination rate of 0.01 for both sexes. Mutation events were assumed not to occur within the genealogy. We simulated 2,000 independent replicates of the genomic region of size 1 Mb for each of the two large genealogies. Hence, if we look at the combined replicates, we effectively simulated the equivalent of 2,000 Mb or almost ∼2/3 of the human genome size.

### 2.2 Genealogical/Coalescent-Based HBD Inference

We inferred HBD status of the probands along the simulated genomic segment using information from both the observed study and coalescent/gene genealogies. The HBD status for a genomic segment was defined as the two haplotype segments finding an MRCA before a threshold time of 30 generations, either in the observed study genealogy or further back in time in the coalescent genealogies. Since the two lineages of interest correspond to a proband's two haplotypes, then they are HBD in addition to being identical by descent. We also examined a continuous measure of genetic relatedness based on the time back until the MRCA for an individuals' two alleles at a locus. For each simulated dataset, both inferred measures of relatedness (status and the continuous measure) were recorded at each of the single-nucleotide polymorphism (SNP) locations in the segment.

### 2.3 Genomic-Based HBD Inference

HBD was also estimated from the probands' simulated genomic segments using the HMM-based approaches implemented in the IBDLD software version 3.13 (Han and Abney, 2011; Han and Abney, 2013) and the FEstim software version 1.3.2 (Leutenegger et al., 2003; Leutenegger et al., 2006; Gazal et al., 2014a). Both methods estimate a probability of HBD at each locus in the segment. For IBDLD, the genomic-based GIBDLD method was used with default values for all parameters except the minor allele frequency cutoff, which was set to 0.02. HBD estimation is influenced by the linkage disequilibrium (LD) pattern of the genomic segment considered. IBDLD incorporates LD in the model used for estimation, while FEstim requires SNPs in low LD. To obtain a low-LD subset for FEstim, the resulting simulated haplotypes were pruned using PLINK version 1.9^1^ (Chang et al., 2015). The pruning was done using the “independent pairwise” option with window size of 1 Mb, step size of 1, and an *r* ^2^ threshold of 0.5. For FEstim, default values were used for all parameters.

### 2.4 Statistical Analysis

All analyses were performed using the R environment version 4.1.1 (R Core Team, 2021).

#### 2.4.1 Description of Study Genealogies and HBD Measures

We calculated descriptive statistics of the two study genealogies using GENLIB. These characteristics include the completeness of the genealogical structures (defined as the number of ancestors present in the genealogy divided by the expected number of ancestors in the complete genealogical structure), the number of lowest common ancestors shared by the parents of the probands, the kinship coefficients among pairs of probands, and the inbreeding coefficients of each proband (Gauvin et al., 2015). The length of simulated segments deemed HBD was obtained from IBDLD HBD probability estimates using a cutoff of 0.5 and was compared to the genealogical inbreeding coefficient using scatter plots. Violin plots were used to compare the distributions of the different HBD measures: FEstim and IBDLD estimates of HBD probabilities and the time to coalescence, in generations, obtained from the observed study and coalescent/gene genealogies.

#### 2.4.2 Correspondence Between Genomic-Based HBD Inference Methods (IBDLD and FEstim)

Correlations between IBDLD and FEstim HBD probabilities were calculated. Specifically, in each study, each simulation replicate ***k*** (***k*** = **1 ... 2,000**) yielded two n×j matrix of HBD probabilities (one for FEstim and one for IBDLD), where n is the number of probands and j is the number of SNPs. Two sets of correlations were obtained for each replicate k . First, the correlation between FEstim and IBDLD probabilities across probands was calculated for each SNP, yielding j correlation coefficients that were then averaged across SNPs to obtain an overall correlation value for each replicate. We refer to this first correlation as the average SNP-wise correlation. Second, the HBD probabilities were first averaged across SNPs, and the correlation between average FEstim and IBDLD probabilities was calculated across the proband, yielding one correlation value for each replicate. We refer to this second correlation as proband-wise correlation. We also displayed the relationship between the average (across SNPs and replicates) FEstim and IBDLD probabilities using scatter plots.

#### 2.4.3 Correspondence Between Genomic- and Genealogical/Coalescent-Based Inference

HBD status inferred from the time in generations to MRCA within 30 generations (as described above) was averaged across SNPs in the simulated genomic region to obtain an estimate of the HBD probability in the region. This estimate was then compared graphically to IBDLD and FEstim HBD probabilities using scatter plots with fitted regression lines. Lastly, we used scatter plots to examine the relationship between genomic HBD probabilities from FEstim and IBDLD and time to coalescence in generations for each k×n×j simulation results. Different HBD probability cutoffs were explored in terms of the generations to coalescence captured by each cutoff. The distribution of generations to coalescence for each HBD probability cutoff was examined using boxplots.

## 3 Results

### 3.1 Description of Study Genealogies and HBD Measures

**FIGURE 1:**
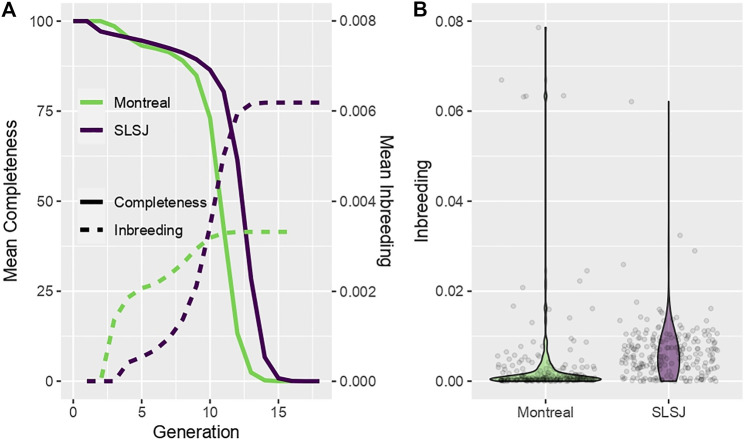
Characteristics of the SLSJ and Montreal study genealogies. **(A)** Average across probands of the genealogical completeness and inbreeding coefficients calculated at each generation. **(B)** Violin plots of the distribution of inbreeding coefficients across probands (points are the inbreeding coefficients of the probands).

The completeness of the genealogies at each generation is shown in Figure 1A for each study sample. The Montreal study genealogy includes 9,095 founders of 227 present-day individuals selected as probands for this analysis and includes 16 generations with completeness of 89% and 73% at the 8th and 10th generations, respectively. Median kinship among the 227 probands is 0.0003, ranging from 0 to 0.016. The SLSJ study genealogy includes 7,608 founders of 226 present-day individuals selected as probands and includes 19 generations with completeness of 91% and 86% at the 8th and 10th generations, respectively. Both studies have low completeness after the 13th generation, corresponding to the time of arrival of the first European immigrants. Median kinship among the 226 probands is 0.005, ranging from 0.00005 to 0.072. For each study genealogy, the average inbreeding coefficients calculated from the genealogical data considering genealogical data at each generation (e.g., for inbreeding calculated at generation 2, all ancestors above generation 2 are removed) are also shown in Figure 1A, while the distribution of inbreeding coefficients calculated at the highest generation with available data is shown in Figure 1B. A few Montreal study participants share common ancestors earlier in the genealogy, leading to higher inbreeding at lower generations (Figure 1A) and some high, outlying values of inbreeding coefficients (Figure 1B). In fact, five participants from the Montreal study are children of first cousins and share additional common ancestors higher up in their genealogy (number of lowest common ancestors ranging from 10 to 24). However, the SLSJ study has higher inbreeding when calculated at later generations (Figure 1A). Inbreeding coefficients are also overall higher in the SLSJ study (Figure 1B). On average, the number of lowest common ancestors shared by the parents of the study participants was 92 (standard deviation, SD = 43, range = 0–306) for the SLSJ study and 44 (standard deviation, SD = 23, range = 0–110) for the Montreal study.

### 3.2 Correspondence Between Genomic-Based HBD Inference Methods (IBDLD and FEstim)

**FIGURE 2:**
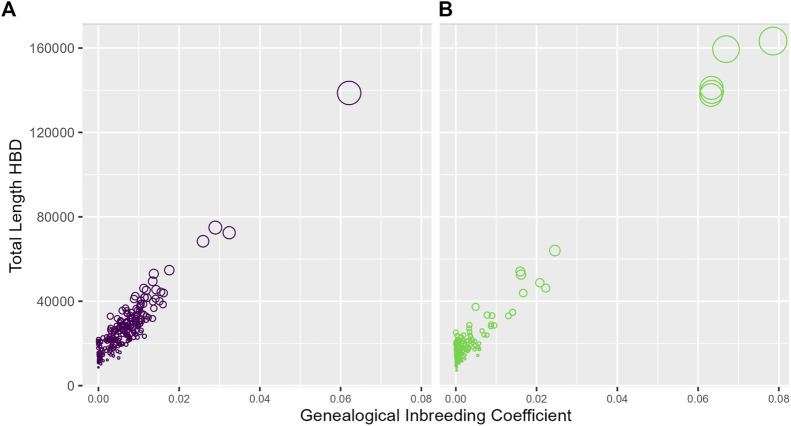
Genomic and genealogical homozygosity by descent (HBD). For each proband, the total length of simulated segments inferred HBD based on IBDLD HBD probabilities of at least 0.5 is plotted against the proband's genealogical inbreeding coefficient. The size of the points is proportional to the number of replicates in which the proband has a segment HBD. **(A)** SLSJ study; **(B)** Montreal study.

**FIGURE 3:**
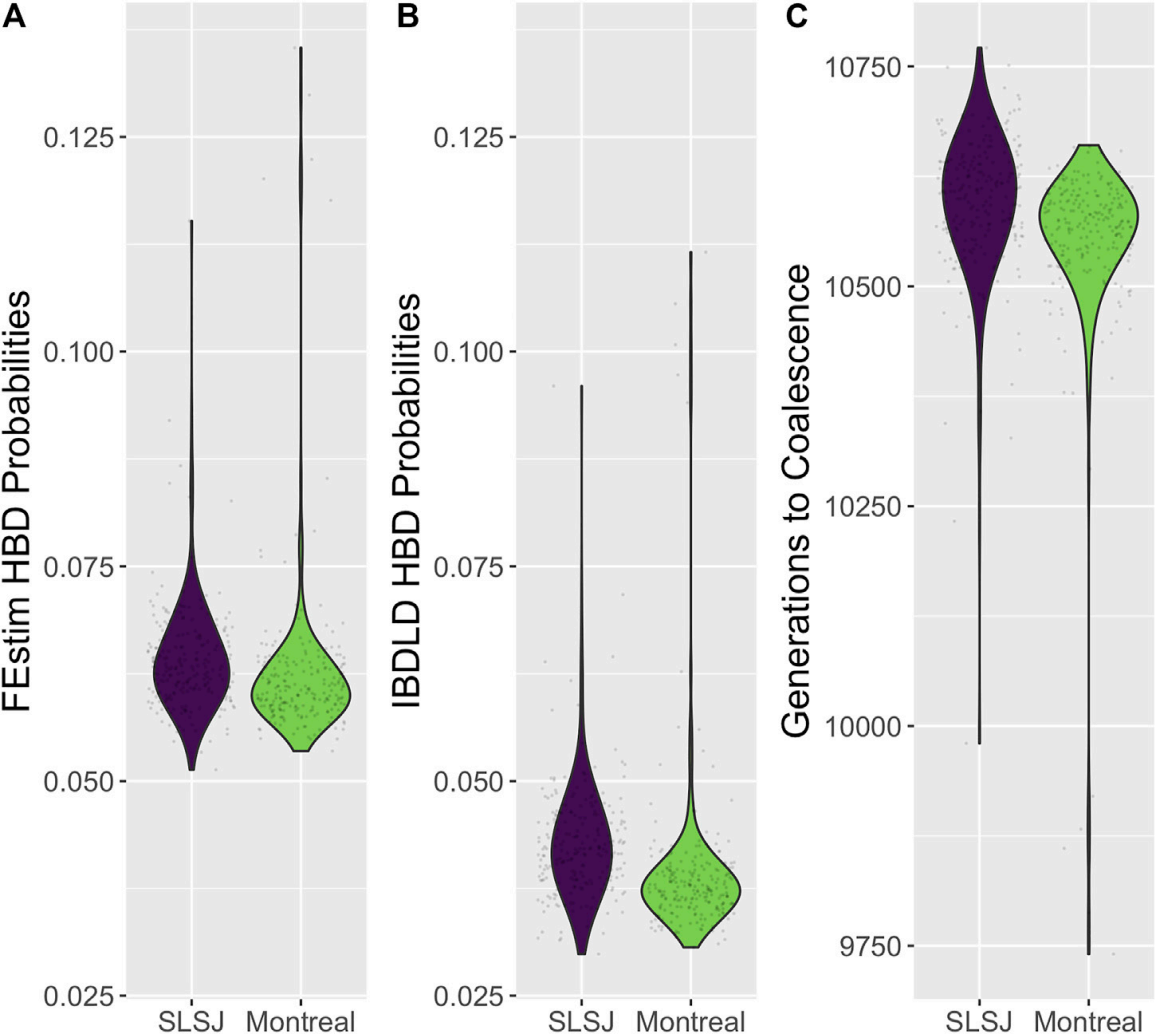
Distributions of inferred homozygosity by descent (HBD). Violin plots of the distributions across probands, averaged across SNPs and simulation replicates, of **(A)** HBD probabilities estimated from FEstim; **(B)** HBD probabilities estimated from IBDLD; **(C)** time to coalescence, in generations, obtained from the observed study and coalescent/gene genealogies.

In Figure 2, for each study, we relate the genealogical inbreeding coefficients of the participants to the total length of their simulated genomic segments deemed HBD across all 2,000 replicates. A SNP is deemed HBD if the probability of HBD estimated by IBDLD is at least 0.5. Summing across simulations mimics the length of a proband's genome that is HBD, which should be highly correlated with the genealogical inbreeding coefficient. The size of a proband's point on the graph is proportional to the number of replicates in which the proband has a segment HBD, which gives an idea of the number of HBD segments in a proband's genome. As expected, the length of genomic segments HBD and genealogical inbreeding correlate well (0.93 for SLSJ and 0.98 for Montreal), and probands with higher inbreeding also have a higher number of HBD segments. The distributions of average HBD probability estimated by IBDLD, FEstim, and generation of coalescence are shown in Figure 3. For the same study data, FEstim HBD probability estimates are overall higher than IBDLD estimates. As noted above, the Montreal study displays inbreeding arising from more recent generations, while the SLSJ study has overall higher HBD.

**FIGURE 4:**
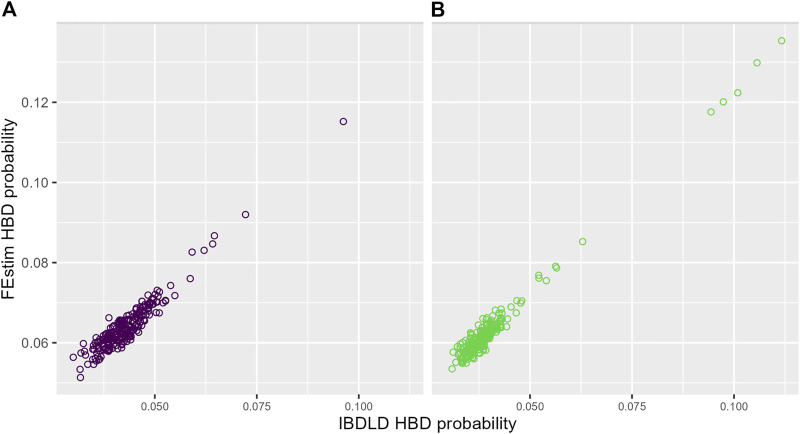
Probability of homozygosity by descent (HBD) from FEstim and IBDLD. Scatter plots of the average (across SNPs and simulation replicates) HBD probabilities estimated by FEstim and IBDLD for **(A)** the SLSJ study and **(B)** the Montreal study.

The relationship between average (over SNPs and simulation replicates) FEstim and IBDLD HBD probabilities is illustrated in Figure 4 for each study. We can see a strong linear relationship between the two HBD measures, and, as also noted in Figure 3, we see that FEstim estimated probabilities are overall higher than IBDLD estimates. We further investigated the correlation between the two methods. Across 2,000 simulation replicates, the mean/median average SNP-wise correlation between IBDLD and FEstim SNP HBD probabilities were 0.7780/0.7813 for the SLSJ study and 0.7694/0.7742 for the Montreal study. When the HBD probabilities were first averaged across the simulated SNPs in the region before calculating the proband-wise correlation, then the mean/median correlations between methods were higher with a mean/median of 0.8991/0.9066 for the SLSJ study and 0.8860/0.8943 for the Montreal study. These results show that there is some variability in estimated HBD probabilities when the correlation is assessed on a SNP-by-SNP basis. However, when the HBD probabilities for probands are averaged across the region, the correlation between the two methods is greater.

### 3.3 Correspondence Between Genomic- and Genealogical/Coalescent-Based Inference

**FIGURE 5:**
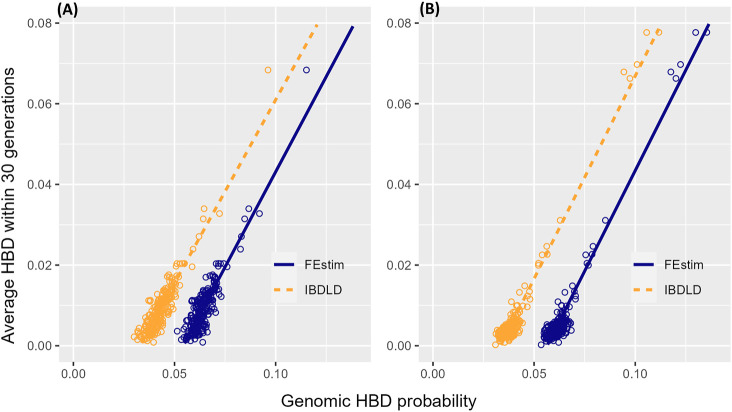
Genomic- and genealogical/coalescent-based homozygosity by descent (HBD). Scatter plots with fitted regression lines of the average (across simulation replicates) HBD probabilities estimated from the observed study and coalescent/gene genealogies and from the simulated genomic segments (by FEstim and IBDLD) for **(A)** the SLSJ study and **(B)** the Montreal study.

It is clear that IBDLD and FEstim differ in their estimated HBD probabilities. To better understand which probabilities might be closer to the true HBD probabilities, we define a gene genealogy-based measure that captures similar information as the HBD probability. For each SNP, we determine if the MRCA of each individual two alleles occur within 30 generations (i.e., this could be within the study genealogy or above but within 30 generations in the coalescent genealogy). Given the small number of generations since the ancestor, the two alleles are very likely to be identical. We then average across the region to obtain an estimate of the HBD probability for each individual in the region. We compare this genealogical/coalescent-based HBD probability to the average HBD probability from both FEstim and IBDLD in Figure 5 by regressing the genealogical/coalescent-based HBD measure on the genomic HBD measure. There is a strong linear relationship between the genomic and genealogical/coalescent-based HBD measures. IBDLD estimates are closer to the genealogical/coalescent-based estimates as indicated by smaller absolute values of the intercepts of the fitted regression lines for IBDLD-compared FEstim. For the SLSJ study, the intercept of the regression line was −0.030 for IBDLD and −0.051 for FEstim, while for the Montreal study, the intercept was −0.034 for IBDLD and −0.056 for FEstim. Hence, in addition to FEstim HBD probability estimates being overall higher compared to IBDLD estimates, FEstim estimates were further from the estimates obtained from the study and coalescent genealogies.

**FIGURE 6:**
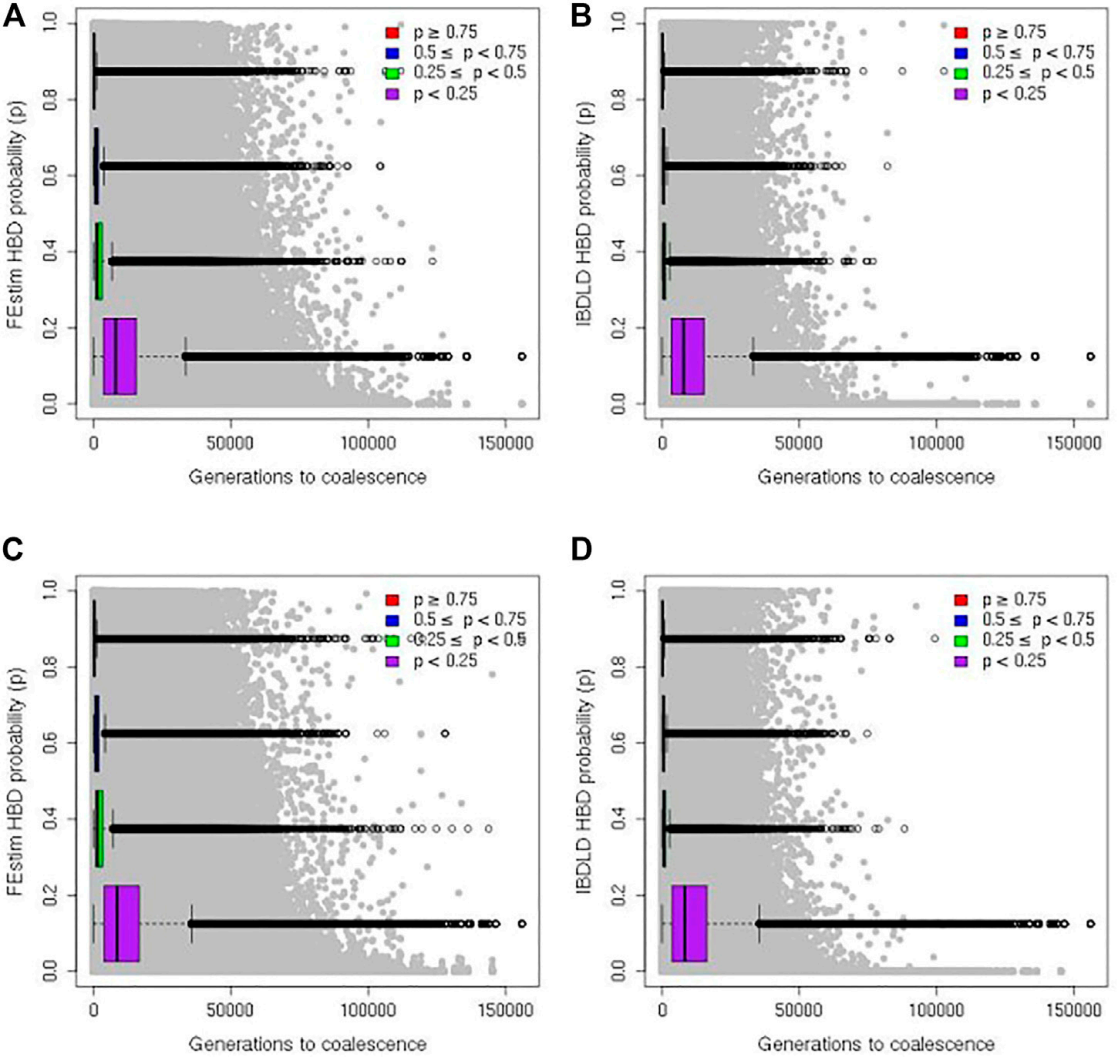
Genomic homozygosity by descent (HBD) and generation to coalescence. Scatter plots of genomic HBD probabilities and generations to coalescence from the observed study and coalescent/gene genealogies. Results from the SLSJ study are shown in **(A)** for FEstim and **(B)** for IBDLD. Results from the Montreal study are shown in **(C)** for FEstim and **(D)** for IBDLD. Boxplots show the distributions of generations to coalescence captured by setting different cutoffs of HBD probability to determine the HBD status of each SNP for each individual.

Finally, in Figure 6, the estimated HBD probability from FEstim and IBDLD for each SNP of each proband is plotted against the generation of coalescence (set to 0 if coalescence occurred within the study genealogy) for all 2,000 simulation replicates. HBD segments coalesced within the study genealogy in 0.63% and 0.33% of all simulation replicates for the SLSJ and Montreal study, respectively. When restricting the study sample to probands with genealogical inbreeding coefficients of at least 0.02 (corresponding to an offspring of parents slightly more related than second cousins), HBD segments coalesced within the study genealogy in 3.8% and 5.2% of all simulation replicates for the SLSJ and Montreal study, respectively.

The distribution of coalescence generation time captured when different genomic HBD probability cutoffs are used is displayed by boxplots in Figure 6. Summary statistics of the distribution of coalescence generation time are shown in Table 1. The figures show a wide range of coalescence time captured by each cutoff. Even with a high cutoff of 0.75, the maximum coalescence time captured can be over 100,000 generations. However, for both studies with both methods (FEstim or IBDLD), the majority of generation times captured are below 1,000 for cutoffs of 0.75 or 0.5, with median generation times captured of ∼160 to ∼290. Times to coalescence captured are overall similar for the SLSJ and Montreal studies. Within each bin of estimated HBD probability, FEstim captures higher time of coalescence compared to IBDLD. Generation times captured are also more variable for FEstim.

**TABLE 1:** Summary statistics of the distributions of the time to coalescence (generations) captured by setting different cutoffs of HBD probability (from FEstim or IBDLD) to determine the HBD status of each SNP for each proband.

HBD prob^a^	SLSJ study				Montreal study			
	Median	IQR^b^	Min	Max	Median	IQR^b^	Min	Max
FEstim								
[0.75, 1]	251	441	0	111,932	282	445	0	145,232
[0.5, 0.75)	959	1,347	0	104,619	973	1,352	0	119,188
[0.25, 0.5)	1,542	2,371	0	123,337	1,541	2,324	0	133,965
[0, 0.25)	8,109	11,957	0	155,952	8,071	11,859	0	145,232
IBDLD								
[0.75, 1]	159	300	0	102,615	180	285	0	92,469
[0.5, 0.75)	461	655	0	82,028	445	594	0	69,544
[0.25, 0.5)	547	1,002	0	76,758	542	914	0	82,197
[0, 0.25)	7,828	11,935	0	155,952	7,775	11,837	0	145,232

a HBD probability cutoffs.

b Interquartile range.

## 4 Discussion

In this study, we simulated chromosomal segments using a coalescent model combined with genealogical data from two study samples from the French-Canadian founder population. We used these simulations to compare two genomic measures of HBD (FEstim and IBDLD) with HBD and relatedness measures based on the study and coalescent/gene genealogies. We found that the genealogies from the two studies had different levels of inbreeding, with the SLSJ study genealogy displaying overall higher levels of inbreeding in concordance with the settlement history of the SLSJ region of Quebec (Roy-Gagnon et al., 2011; Gauvin et al., 2014). Interestingly, the Montreal study had a few outliers with high inbreeding and a non-negligible overall level of inbreeding, indicating that even when recruiting participants in an urban region of Quebec, high levels of relatedness and inbreeding can result in the sample. Considering the effect of inbreeding on the study results would be relevant, and using methods that take into account hidden relatedness is necessary. This could be done using genomic or genealogical estimates of relatedness incorporated into mixed regression models (Zhou and Stephens, 2012; Loh et al., 2015; Ziyatdinov et al., 2018). The total length of segments HBD (obtained with IBDLD) correlated well with the genealogical inbreeding coefficients in both studies, as previously documented (McQuillan et al., 2008; Roy-Gagnon et al., 2011).

In the two studies, the FEstim and IBDLD genomic-based measures of HBD were well correlated when averaging across the simulated chromosomal region but less so on a SNP-by-SNP basis. The correlation between the two methods was also slightly higher in the SLSJ study with higher inbreeding. Hence, the two methods could lead to different conclusions on the HBD status of specific SNPs, especially in study samples with lower inbreeding. Both genomic HBD measures correlated well with the genealogical/coalescent-based measure in both study samples, although IBDLD estimates of HBD probabilities were closer in values to the genealogical/coalescent-based estimates. This could impact HBD status inference from FEstim compared to IBDLD. The difference between the two methods could be due to the fact that IBDLD uses all SNPs in estimating the HBD probabilities since it models LD and thus does not need to restrict the analysis to a subset of SNPs in low LD, as required by FEstim. Gazal et al. (2014b) found that FEstim applied to a sparse set of SNPs in very low LD (SNPs that have a pairwise genotypic correlation *r* ^2^ > 0.01 within a 50-marker window were removed) or averaged over several subsets in low LD (of one SNP selected every 0.5 cM) performed as well as or better than HMM modeling of LD when estimating the inbreeding coefficient using ROHs. In contrast, Han and Abney (2011) used a sparse map of one marker every 1 cM and found that their HMM modeling LD was superior in estimating the proportion of identical alleles shared by descent. These studies did not directly evaluate the correspondence between genomic-based estimates of HBD probability and HBD probability obtained from the study and coalescent/gene genealogies. When using FEstim, we selected LD-pruned subsets of SNPs based on Howrigan et al. (2011) with less stringent LD pruning than Gazal et al. but more stringent than Han and Abney. LD pruning could affect the difference that we observed between the FEstim and IBDLD estimates of HBD probabilities. Since the IBDLD genomic-based estimation of HBD always incorporates LD, it is not meant to be used on LD-pruned subsets of SNPs. Thus, we could not compare IBDLD and FEstim on the same set of SNPs in low LD.

In defining the genealogy/coalescent HBD measure used in the comparison with the genomic HBD probability estimates, we considered whether the MRCA occurred within 30 generations (i.e., it could be within the study genealogy or within 30 generations) for each SNP and averaged over the region. Results comparing this genealogy/coalescent HBD to the genomic HBD probabilities (averaged over simulation replicates) shown in Figure 5 were very similar to those obtained when considering whether the MRCA occurred within the study genealogy only instead of within 30 generations (data not shown). Considering 30 generations captures only little additional variation for low HBD probabilities, yielding low average HBD probabilities close to 0 instead of equal to 0 (data not shown). This reflects that most of the variability in inbreeding is due to recent inbreeding (Keller et al., 2011) captured by the deep genealogical data available in both studies.

When using genomic estimates of HBD probabilities, a cutoff can be used to determine if a SNP is HBD or not. A probability cutoff of at least 0.5 is the default in the IBDLD software. Using a cutoff is useful in order to infer HBD segments or to assess associations between HBD at specific genomic regions and health-related phenotypes (Ceballos et al., 2018). It is thus helpful to consider the time to MRCA captured by different cutoffs. Our results indicate that a cutoff of at least 0.5 in IBDLD probability mostly captures generations to coalescence time under 500, while a cutoff of 0.75 mostly captures generations to coalescence time under 200. Generations to coalescence captured were higher for FEstim, likely reflecting the higher HBD probability estimates for FEstim overall. As discussed above, LD-pruning parameters may influence these results. Gazal et al. (2014b) recommend considering several sparse subsets of SNPs, but this approach has been evaluated in the context of genomic inbreeding coefficient estimation and not directly for the genomic HBD probability estimates. More studies comparing different LD-pruning approaches would be helpful. At all cutoffs, generations to coalescence captured is quite variable on a SNP-by-SNP basis. Considering segments of specific lengths may reduce this variability. Based on a subset of 25 replicates of the SLSJ simulations (yielding over one million SNPs), the correlation between length of HBD segments within which SNPs are located and generation time to coalescence were −0.23, −0.26, and −0.29 for IBDLD HBD probability cutoffs for determining HBD of 0.5, 0.75, and 0.9, respectively. This indicates that SNPs capturing recent coalescent events are located in longer segments. Considering a cutoff of IBDLD probability of at least 0.5 to determine if a SNP is HBD, 75% of SNPs located in segments greater than 750 kb captured generation times to coalescence of 30 years or less, while 98% of SNPs located in segments smaller than 25 kb captured generation times to coalescence over 500 years.

Our study has some limitations. First, the genealogical data from the two French-Canadian studies are not complete. The incomplete genealogical links could affect our simulation results in terms of the generation time to coalescence. However, since our simulations are based on these incomplete genealogies, the missing genealogical links are taken into account in the simulation results. Second, it would be interesting to vary the parameters used for the coalescent simulations. Third, using genome-wide SNPs instead of a 1-Mb segment would allow comparison with a wider range of HBD detection methods (e.g., those based on ROHs) and would more closely reflect studies of HBD, which are typically genome-wide. In this study, we prioritized computational feasibility to be able to trace back the generation of coalescence in a large number of simulation replicates. Finally, studying more parameters and conditions (e.g., missing genotypic data) for LD pruning or the HMM LD modeling would provide more guidance on the application of these analysis tools.

In addition, the two study genealogies used in our simulations come from participants selected based on disease status. Thus, our results may not generalize to samples obtained without ascertaining on a disease status. However, the two studies considered in these simulations investigate complex diseases influenced by many genetic and environmental factors. Hence, although participants were ascertained based on their disease status, we do not expect to see a large difference in overall (genome-wide) inbreeding levels in the study samples compared to the general French-Canadian founder population. Indeed, the kinship and inbreeding levels observed in the two studies correspond to those observed in Roy-Gagnon et al. (2011) in the SLSJ and Montreal sub-populations. This study examined kinship and inbreeding in sub-populations of the French-Canadian founder populations that were not selected based on disease status. Moreover, in the Montreal study, kinship levels within and across disease or control groups were similar. Within groups, the median genealogical kinship coefficients ranged from 0.00027 (interquartile range, IQR = 0.00026) in the glaucoma group to 0.00031 (IQR = 0.00028) in the control group, while across group, the median genealogical kinship coefficients ranged from 0.00026 (IQR = 0.00027) for AMD–glaucoma pairs of individuals to 0.00029 (IQR = 0.00028) for AMD–control pairs. However, in both studies, we would expect IBD/HBD sharing measured locally around a disease-causing genetic variant to be different in affected individuals. In our simulations, we did not consider disease-causing variants. We thus expect our results to be similar in cohorts not ascertained based on a disease status with similar levels of inbreeding. We also expect our results to be similar in other founder populations with kinship/inbreeding structures that are not too far from the two study genealogies included in our simulations. The two studies that we considered include different genealogical structures with different levels of kinship and inbreeding. Our results are overall similar across the two studies.

In summary, our simulation results provide insight for the interpretation of genomic estimates of HBD in a large founder population. We studied two genealogical datasets from different study designs yielding different levels of inbreeding. These differences in inbreeding led to different genomic estimates of HBD, which correlated well with genealogical/coalescent HBD. Generation time to coalescence captured by genomic HBD estimates was similar in the two studies. Time to coalescence captured was very variable when considering each SNP separately, but SNPs in longer segments were more likely to capture recent time to coalescence, as expected. Our study suggests that estimating the coalescent gene genealogy from the genomic data (Burkett et al., 2013; Burkett et al., 2016; Karunarathna and Graham, 2019) and combining these estimates with observed genealogical data when available could provide valuable information for HBD inference.

## Data Availability

The raw data supporting the conclusion of this article will be made available by the authors, without undue reservation.

## Footnotes

1 Purcell, S., and Chang, C. PLINK 1.9. Available at: www.cog-genomics.org/plink/1.9/.
